# Supporting Health and Social Care Students Stay and Stay Well: A Conceptual Framework for Implementing Integrated Care Into Higher Education

**DOI:** 10.5334/ijic.7772

**Published:** 2025-01-23

**Authors:** Louise Grant, Lisa Bostock, Caroline Reid, Nasreen Ali, Fiona Factor

**Affiliations:** 1 Department of Education and Training, Tavistock and Portman NHS Foundation Trust, 120 Belsize Lane, London NW3 5BA, UK; 2 Faculty of Health and Social Sciences, University Square, UK; 3 University of Bedfordshire, Luton, LU1 3JU, UK

**Keywords:** integrated care, higher education, inter-professional education, pedagogy, emotional resilience, inter-professional reflexivity

## Abstract

Integrated care demands a workforce that is confident, capable and compassionate. This is dependent on a willingness to work inter-professionally and understand the roles, standards and values of other professional groups. However, there are few examples of integrated care initiatives within higher education that aim to build the knowledge and skills required to support effective integrated, people-centred care.

While satisfying, working in the helping professions is emotionally challenging and for students’ these challenges are often underestimated. Some students struggle through their studies with many failing to complete and others drop out in the early years of their careers. Understanding what supports students to thrive in their professional roles is essential to retention of a highly skilled integrated workforce.

To address this challenge, this paper outlines a conceptual framework designed to promote a pedagogical environment focused on creating the conditions for integrated working. The framework is based on the “student lifecycle”, from starting to see the benefits of a career in the helping professions, developing a sense of belonging through to thriving and succeeding as future practitioners. It outlines how students are supported to develop emotional resilience, inter-professional empathy and reflexivity to help them stay and stay well in their careers.

## Lessons learned

- Despite the constraints of current statutory and professional regulatory bodies requirements for professional education, opportunities exist to promote inter-professional learning via creative and innovative ways of fostering connections
- Inter-professional empathy and reflexivity are key to creating the conditions for mutual respect in health and social care students
- Supporting students begin, belong, thrive and succeed at each stage of their university career and beyond into professional practice provides a framework for prioritising the needs of a diverse group of students
- For integrated care curricula to be truly successful, education colleagues need to be included in design and delivery
- If students are to be prepared to deliver integrated care, universities need to be part of a wider conversation about how “true” inter-professional education can be achieved

## Introduction

In line with the World Health Organisation (WHO) framework on integrated, person-centred care, governments across the globe have been developing or strengthening their approaches to achieving an integrated continuum of care [1]. The WHO framework aims to position people at the heart of health and social care systems and improve access to holistic care for people experiencing a wide range of long-term health issues. Across all four countries of the UK, there has been a focus on strengthening governance and accountability via legal requirements to collaborate using joint governance structures and pooled budgets [2]. In July 2022, England introduced a new place-based initiative to design and deliver local integrated services via statutory health and care partnerships, governed by an integrated care board (ICB) [3].

At the root of integrated care is the importance of professional collaboration and inter-professional working. However, such terms are subject to debate and conceptual confusion within the literature [4]. A recent systematic review, while providing a definition of inter-professional collaborative working as an ideal state where professionals work together to provide holistic care, goes beyond this to describe the key features that mark out success in this sphere. They propose that inter-professional practitioners need to be boundary spanners and space creators who are willing to be flexible and seek out others to assist the delivery of care [5].

In addition, integrated care requires those involved with its planning and delivery need to prioritise the “patient perspective as the organising principle of service delivery” [6]. However, in a scoping review of the evidence on education, training and workforce development in integrated care, Barraclough et al. argue that the current health and social care workforce are ill-equipped to provide holistic integrated care [7]. While widely recognised that the delivery of such care requires a compassionate and inclusive workforce, there are few examples of integrated care initiatives within higher education that aim to build the knowledge and skills required to support effective integrated, people-centred care.

Unlike Canada, Belgium and Serbia, there is no comprehensive framework for inter-professional education (IPE) in the UK [8,9]. IPE “occurs when students from two or more professions learn about, from and with each other” to improve their ability to communicate, collaborate and care effectively for people using services [10]. In the UK, qualifying education and training are regulated via a series of profession-specific health and social care bodies. These include the Nursing and Midwifery Council (NMC), Social Work England (SWE) and the Health and Care Professional Council (HCPC) who regulate 15 profession groups including, paramedics, physiotherapists and occupational therapists. While they all require students to have elements of IPE these tend to be additions to the profession specific curricula rather than truly cross professional.

In addition, there is widespread concern that students in the helping professions are not well prepared for the emotional demands and realities of their chosen careers [11] leading to attrition either during their training programme or in their preceptorship, with stress and lack of support cited as the key drivers for exit [12,13,14]. This is compounded by the challenges of working within an integrated care system that brings together different professional groups from well-established training, practice and service silos [15]. Yet, little is known about the extent to which the qualifying curriculum reflects the need for students to develop the inter-professional skills required to manage the emotional demands of practice. This includes enhancing their resilience to stress, building their self-confidence and sense of professional identity to enable delivery of compassionate and inclusive, integrated care.

Educating competent, compassionate and resilient practitioners for the future requires a multifaceted approach, one which enables professionals to work in an inter-disciplinary way and in an integrated environment [16]. There have been calls for the development of a signature pedagogy for inter-professional health and social care education which shares a value base and commitment to developing collaboration and inter-disciplinary holistic care [17]. While not proposing a signature pedagogy, this paper outlines a conceptual framework structured to address the gap in the current curriculum by designing a pedagogical environment focused on creating the conditions for integrated working. The framework is designed to promote a culture of respect for differing professional perspectives by strengthening inter-professional reflexivity. That is, the ability to interrogate the role of self, cognition and emotion via dialogue with others within the integrated system to surface biases and assumptions by working through them together to create shared meaning, understanding and inter-professional empathy [18,19]. Integral to the framework is a focus on developing a learning culture that promotes self-confidence, emotional resilience and self-care to support emerging health and social care professionals to “stay and stay well” within their chosen careers [20].

## What competencies are required to deliver integrated care?

Achieving the transformation required by integrated care, the following skills and competencies have been identified. They include teamwork and inter-professional communication, person-centred care, patient empowerment, knowledge of the needs of people within their communities and health promotion strategies [7,21,22,23]. Critically, the WHO identify resilience as essential to the effective delivery of an integrated, people-centred approach [1]. Ensuring that the skills and competencies required for integrated care are taught and developed, in part depends on embedding them within formal education and training systems via the pre-qualifying curriculum [24].

However, the current qualifying curriculum has been criticised, with several authors calling for an overhaul of existing uni-professional educational programmes. These programmes are described as inflexible, linear, and not fit for purpose for the implementation of effective integrated working [7,25,16]. Rather than training professionals in silos, it is recognised that there needs to be a curriculum culture shift to multi-disciplinary training by re-organising the delivery of professional education. Nonetheless, the current professional statutory regulatory body requirements and the need to enable students to fully understand their chosen professional knowledge and skills base makes this difficult. Furthermore, uni-professional training is an important element of the socialisation and safe practice of a professional [24]. Creating a truly multi-disciplinary curriculum is, therefore, likely to be challenging given the short duration of training and the profession specific requirements needed for successful completion of a course.

Nevertheless, the importance of developing an environment that will cultivate the required competencies and training that supports the development of a competent workforce able to deliver integrated care is key [24]. Drawing on the evidence identified via their scoping review, Barraclough et al. specifies the skills and competencies required for integrated working and summarise some suggested curriculum principles for effective IPE [7]. They argue that it is essential to:

- engage faculty teaching staff who convey joy in their work and provide trainees with education around work-life balance, self-reflection and self-improvement
- create a working environment that values wellness and creates a climate of respect and work-life balance
- incorporate simulation-based scenarios using actors from the local community with lived experiences (7).

Based on these principles this paper sets out a conceptual framework aimed at ensuring students continue to be educated in their own specific areas of expertise while also preparing them for integrated working.

## Beginning to belong, to thrive and succeed: a conceptual framework for implementing integrated care into higher education

In 2022, the University of Bedfordshire was awarded a grant to work with its newly established local integrated care board (ICB). The award established the University as an anchor institution, providing research and developmental support to regional partners to address health inequalities through the creation of an effective integrated care system. The area it covers is home to 1 million people, with the number of people aged over 85 expected to double by 2035. When compared with the rest of the UK, there is a significantly higher proportion of people from Asian ethnic groups (13% compared with 7.5% in the general population) and White groups other than those from the UK and Ireland (8% compared with 4.5% nationally). Around 120,000 (13%) people live within areas identified as among the top 20% most deprived in England [26]. This means that the workforce needs to both reflect the diversity of the population it serves and have the competencies required to respond effectively to the needs of a diverse population who can face significant health inequalities.

**Figure 1:**
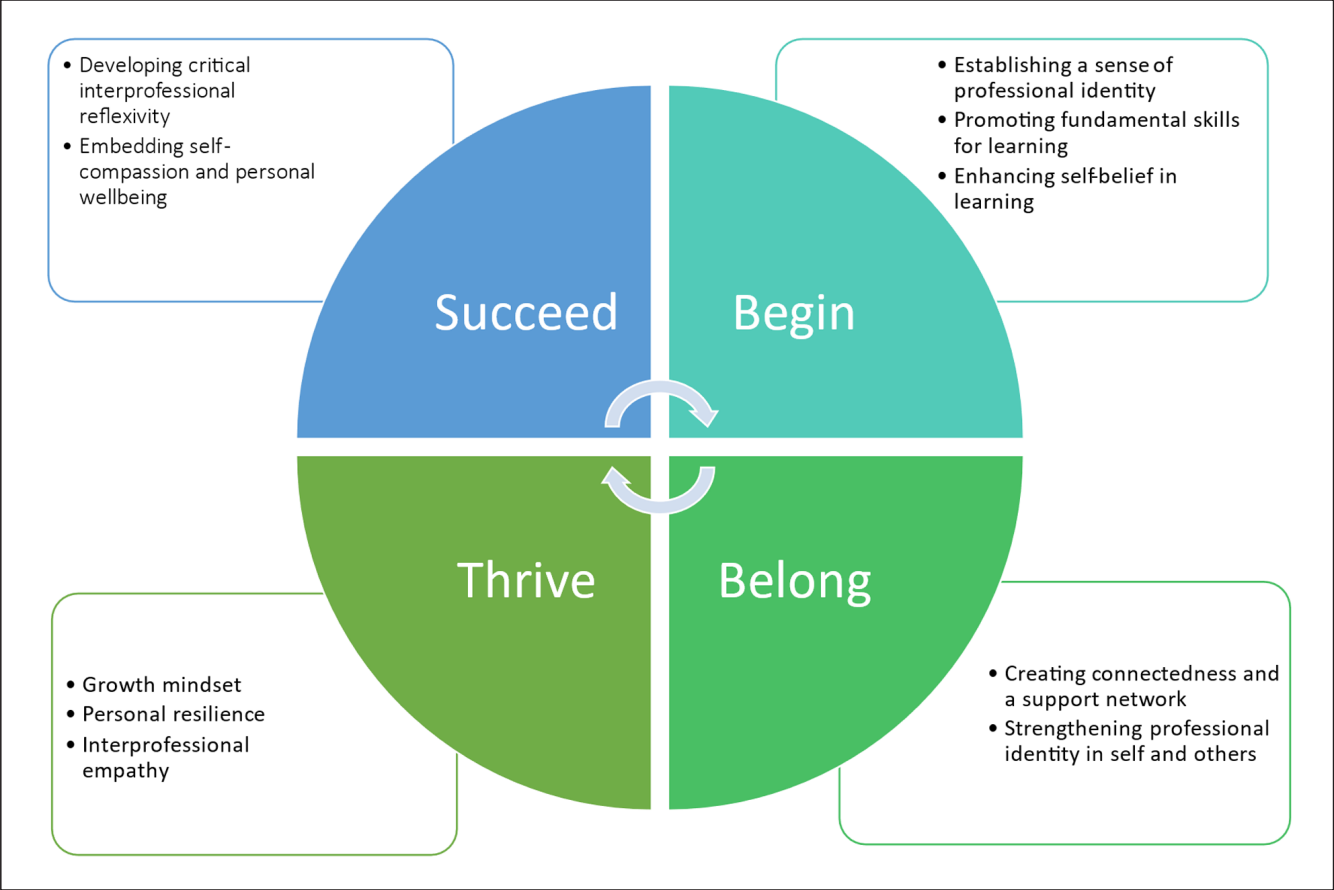
Conceptual framework to embed integrated care into the curricula.

To meet this challenge, senior leadership within the Faculty of Health and Social Sciences (HSS) convened a series of sessions with curriculum leads and researchers from across professional directorates to review the Faculty’s approach to inter-professional teaching and learning. It was agreed to re-structure the IPE curriculum around a conceptual framework based on the “student lifecycle” [27]. The framework (Figure 1) aims to capture and support student experience from preparing for university to final qualification and beyond into professional life. It is organised around the themes identified by the UK-based Student Futures Commission [28] of begin, belong, thrive and succeed (see Figure 1). The implementation of these concepts has been informed by research, teaching and learning initiatives undertaken by the authors to ensure students are equipped to manage the challenges of delivering compassionate and inclusive care centred around the needs of the person. They are designed to promote good communication, collaboration, and the ability to care effectively for people using services [10]. The following sections explore these key concepts and how the framework has been developed.

## Beginning to belong within the health and social care professions

A successful beginning at university requires an understanding of students’ needs and previous experiences. Many students come to our University as first-generation scholars. These students tend to be from lower-income socio economic groups and in line with the wider population of “first in family” graduates, are more likely to be from Black, Asian or other minoritised backgrounds [29]. This cohort of students bring a wellspring of “cultural wealth” to their studies, with many having developed problem-solving skills and personal resilience to succeed in dealing with experiences of structural inequalities [30]. They also have the potential to deliver more inclusive and culturally responsive care. However, they can also enter higher education feeling self-conscious and out of place and often require extra support to adjust and settle into their studies [24].

Improving the knowledge, perceptions and possibility of success in a career in health and social care, the University has developed a Collaborative Targeted Outreach Programme (CTOP) [31]. CTOP is a culturally competent intervention that uses a range of approaches, including applied theatre and immersive methods to engage in dialogue with diverse communities and prospective students about the benefits of a career in integrated care. The intervention is based on the premise that if you see others that look and sound like you being successful, it challenges barriers, improves self-belief and confidence as well as raising consciousness about the possibility of belonging within the helping professions.

Developing a culturally competent learning culture where students are viewed as trailblazers who can use their previous experiences as assets, helps support a sense of student identity and avoid feelings of alienation within the higher education system [24]. This is supported by a summer school focused on developing self-confidence, self-efficacy and curiosity to prepare them for learning at university. In addition, this is underpinned by teaching and learning key academic skills such as maths and English, to support them in their academic work before their formal course commences [26]. Beginning well is precursor for belonging, given students who report having had a good beginning are three times more likely to feel they belonged at university than students who did not settle well into their studies [27].

## Belonging: building confidence, connection and professional identity

Once students have started their professional courses, staying in education is in part dependent on creating a culture of belonging [33]. Students who feel they belong at university are more self-confident, feel more connected and report better wellbeing [28,35]. A sense of belonging is positively associated with higher grades and higher student engagement [30]. Therefore, belonging is essential to retaining students who have the skills and competencies required for integrated working [24]. For students from underrepresented groups, developing a culture of belonging is particularly important to ensure that they feel included and supported to thrive and succeed in their studies [37].

The four foundations of belonging – connection, support, inclusion and autonomy – have been integrated into the pedagogic framework for health and social care students [27]. This initiative, Belong@Beds (B@B), has been co-created with students and aims to improve inclusivity, social support and student wellbeing [32]. As part of the strategy, a room has been designated as communal social learning space for health and social care students, providing students and staff with a place to meet, share ideas and reflect on learning together. Student Inclusivity Network Groups (SING) have also been introduced for those who experience a range of protected characteristics, for example the Black Student Network. Together these aim to improve connection with and between students undertaking professional courses. Finally, students are encouraged to seek support for their mental wellbeing at an early stage from teaching staff have been trained as mental health first aiders.

## Thriving: Developing a growth mindset to foster inter-professional learning and respect

As students move through their education, thriving in their studies is determined by a growth mindset [33]. People with a growth mindset believe that ability is not just dependent on a fixed intelligence but that their skills and abilities are developed through their motivation for learning [33]. Associated with both self-confidence and self-efficacy, a growth mindset can counter imposter syndrome often experienced by students from underrepresented groups [34,40]. Spreitzer et al. defines thriving at work as the “psychological state in which individuals experience both a sense of vitality and a sense of learning at work” [36].

Thriving is enabled by teaching staff that convey joy in their work and promote learning environments that encourage inter-professional reflexivity and self-improvement in preparation for integrated working [5]. To create this sense of thriving and enjoyment, Schwartz Rounds have been introduced for staff and students as part of an initiative to embed Rounds within the HEI sector [42]. Rounds are a licensed intervention, starting originally in the USA and conceived to promote humanity and compassion in the way we treat our colleagues and therefore people using services [37]. Rounds are a multi-disciplinary, reflective forum where students and staff can come together to discuss the emotional impact of their work and training. Rounds are facilitated by staff and start with a panel of 2–3 student storytellers who share their experiences of topics such as, “a fear that I have overcome”. Social work and nursing academics have trained as Rounds facilitators, working together for the first time to support students craft their stories, in ways that will create emotional resonance with audience participants from across professional courses. This has enabled students to participate in a unique multi-disciplinary, reflective space, structured to specifically to enable connections and understanding to develop at a deeper, emotional level that reveals the person behind the professional. While aimed at helping students develop a growth mindset they have also enabled staff to reconnect with the rewards of working in the helping professions. Evaluation feedback from both staff and students has demonstrated the value of creating such connections across disciplinary boundaries through sharing, what are often inspiring stories of overcoming fears or lack of professional confidence to provide people with personalised care, sometimes in emergency situations. Rounds have been shown to reduce stress, improve connectedness and promote inter-professional empathy among regular participants, including students [37,43].

To further capture the imagination of students, a programme of simulations are being developed to embed inter-professional teaching and learning. Developed between staff and students, a simulation focused on a child protection incident that had taken place at the family’s home involved a range of professional groups, including midwives, social workers, community nurses, paramedics. The simulated incident was attended by a “simbulance” which is fully functioning ambulance used for the purpose of teaching of paramedic students. In line with the experiences of others developing inter-professional teaching and learning initiatives [44], academics initially hesitant to embrace new pedagogic practices, engaged with enthusiasm, owning their own expertise but also willing to see different perspectives on an integrated care delivery. Interestingly, the roles and priorities of the different professionals involved in developing the scenario became evident. The midwifery academics were coaching the students to focus on the health and well-being of mother and baby, the paramedics on the emergency in front of them and the social workers in ensuring the safety and well-being of all the children in the household was prioritised. Co-creating the simulation generated much discussion as well as laughter as differences in professional talk, understanding and practice were navigated in a safe and fun way. While both Schwartz Rounds and simulations aim to encourage a thirst for inter-professional learning and respect via creative and innovative ways of creating connections, it is recognised that the inclusion of teachers, health visitors and other integrated care professionals are essential to further simulation exercises.

## Succeeding: enhancing personal resilience and inter-professional reflexivity

Succeeding can be defined by staying and stay well to deliver integrated care. This is dependent on working within an environment that values wellness, work-life balance and creates a climate of respect for other professionals [5]. While challenging, working in the helping professions is generally experienced as rewarding and associated with occupational success and well-being [38]. To preserve this sense of personal accomplishment is in part, determined by successfully managing emotionally charged situations to maintain professional practice standards and protect personal wellbeing.

Emotional resilience is the ability to positively adapt and grow from adversity and challenges and is a key resource for those working in the helping professions [39]. Resilience is conceptualised as a process whereby a person’s ability to positively respond and grow from difficult situations is enhanced by wider supports and organisational culture. It has been described as an umbrella term as concepts of belonging, thriving, self-confidence and self-efficacy are all positively linked with being resilient in the face of challenges.

Building on the work of Grant et al. [45], a web application has been developed to help students and qualified staff in integrated care improve their personal resilience. The Integrated Systemic Organisational Resilience Toolkit (iSort) is an accessible, research-informed diagnostic survey and set of associated strategies designed to build and sustain personal resilience. Strategies include building personal wellness action plans and fostering flexible coping strategies. With a focus on a positive psychological approach, it aims to improve wellbeing and emotional resilience with the ambition to improve retention and promote flourishing and success.

At all stages of professionals’ careers, from newly qualified to experienced senior leadership roles, it is important to recognise and address the emotions that are experienced in practice. Uncertainty in thinking and reasoning is crucial in allowing professionals to engage with the complexities of practice, yet can create anxiety, both for individual practitioners but also among the wider inter-professional network [40]. Reflection is an important self-protective mechanism which can support the competencies that underlie emotional resilience [9].

Within the integrated care context, it is important to foster forums that promote reflection and inter-professional reflexivity to challenge our taken for granted professional positions and build effective working relations [19]. In the next part of our strategy, we will be introducing two new initiatives. The first is focused on inter-professional supervision in health and social care. Building on the work of Bostock et al., this post-qualifying course aims to support delivery of effective supervision across disciplinary boundaries to promote person-centred care [41]. Effective reflective supervision is associated with improved quality of practice, worker wellbeing and staff retention [42,48].

A second initiative is focused on building inter-professional reflexivity to enable the possibility of sharing dilemmas and challenges with colleagues from across the integrated care continuum and further promote anti-racist practice. Inter-professional reflective practice groups (RPGs) have been introduced for senior leaders in health, social care and education. RPGs are facilitated, structured reflective forums that are designed to provide emotional containment for the anxiety inducing effects of working with a complex, integrated working landscape. The enable generation of multiple perspectives, and hence multiple solutions on the problems presented for discussion [47]. RPGs have been found shown to promote inter-professional collaboration, lower intolerance to uncertainty and reduce professional anxiety [43]. Together these initiatives aim to create a climate of mutual respect through “thinking aloud” [49] to manage the emotionally charged nature of work in the helping professions and challenge thinking that may be based on stereotypes, bias, or unconscious racist assumptions.

## Discussion

Globally, it is estimated that there will be a shortfall of 10 million healthcare workers worldwide by 2030 [45]. In addition, increasing ethnic diversity and an ageing population will lead to greater demand for holistic and inclusive person-centred care [1,46]. It is therefore imperative that qualifying education and training programmes prepare students for the realities and possibilities of working within integrated care.

Despite this global need there is, to date, limited research on how pre-qualifying curricula can prepare students for the delivery of high-quality integrated care [50]. Staying and staying well to deliver integrated practice is a priority and preparation needs to commence during the qualifying years. However, restrictions within current professional training means that, while trainee professionals have some inter-professional learning, overall, they are taught separately [44,51]. To address this challenge, this paper has outlined a curriculum framework which from the point of inception, focuses on the development of competencies and skills required for integrated care. This is based on a “student lifecycle” approach and designed to provide incremental support at each stage of their university career and beyond into professional practice. It aims to enable them to successfully:

- Begin: by fostering self-belief, self-confidence and curiosity pre-entry
- Belong: by building confidence and connections once at university
- Thrive: by developing a thirst for inter-professional learning and self-improvement
- Succeed: by enhancing emotional resilience, inter-professional reflexivity and anti-racist practice.

It is based on the following proposition: if health and social care students begin well, have a sense of belonging to their professional course and are provided with the strategies to enhance emotional resiliency and wellbeing that they will thrive and succeed and will be better placed to provide compassionate and inclusive care.

The framework is founded on the assumption that providing holistic, person-centred care can be both rewarding and emotionally challenging. Many students in the helping professions find their qualifying education and training demanding. A significant number struggle through their studies, sometimes failing to complete while others drop out in the early years of their careers. High drop-out rates adversely affect our ability to meet the workforce demands and contribute to the need for an increase in the health and social care workforce. An endless cycle of attrition is created, further exacerbated by experienced workers opting for early retirement post pandemic [12]. Addressing this issue via the pre-qualifying curricula is essential given the international crisis of recruitment and retention of health and social care practitioners [45].

In line with the work of Barraclough et al., the curriculum aims to create the conditions for learning that promote emotional resilience, wellbeing and work-life balance [7]. While not developing a signature pedagogy, this paper outlines a curriculum framework designed to create the conditions for effective inter-professional collaborative working. An emphasis therefore, on promoting mutual respect, inter-professional empathy and reflexivity during training and into professional practice is prioritised. This is further scaffolded via strategies on developing skills for emotional wellbeing is integral to this curricula model. In addition, the focus on creative and innovative recruitment methods to widen participation and build self-confidence and belonging has the potential to improve integrated care understanding and practice. Combined with prioritising the acquisition of flexible coping strategies and tools to create wellness action plans, students will be enabled to better manage the demands of their future careers which in turn, may lead to greater retention. Generating inter-professional collaboration in a university setting has its challenges. There is an implicit “competition” among academics as to whose experience, knowledge and skills should be prioritised and this can only be overcome by the team reflexivity described above and in the helpful inter-professional literature [49]. In addition, and in line with other research in this field [49] personal motivation, enthusiasm, a shared vision and a genuine regard for diverse professional skills and knowledge is essential.

A limitation of this work, so far, has been a lack of involvement with students who are qualifying as teachers. Calls have been made for greater integration with teacher education which is particularly important for effective integrated care in the provision of support and protection to children and young people [47]. Currently many Schools of Education sit outside those educating health and social care workers presenting a barrier to joined up working with governance often siloed preventing collaboration. These practical obstacles are not insurmountable but may need to be tackled by statutory regulatory bodies for professional education mandating more explicitly the need for inter-professional learning if pedagogic practices are to be changed.

Despite the challenges of implementing a truly integrated curriculum, doctoral research is currently underway with the aim of implementing a greater emphasis of integrated working across all the pre-qualifying curricula at the University [52].

## Conclusion

There is global acceptance of the need for an integrated care system to provide holistic care and services for people experiencing a wide range of long-term health and well-being issues. While much is written is about policy and practice initiatives, universities appear to be a missing partner in integrated care delivery, yet are integral part to the integrated care system. This starts with a qualifying curriculum that is premised on inter-professional teaching and learning that promotes a common purpose to provide person-centred care across disciplinary boundaries.

Collaboration in higher education would be aided by University departments being organised to facilitate integration with education, health and social care courses located in the same Faculty. While in its early stages of development, it is hoped that the adopted approach will increase diversity, reduce attrition during training and promote greater inter-professional empathy, communication and collaboration for effective inter-professional practice. It proposes a curriculum framework that moves away from the constraints of uni-professional curricula until wholesale reform can be achieved across inter-professional education. Evaluation of the curricula’s overall effectiveness and the contribution of the individual components are being undertaken but the process of developing the framework, has provided opportunities for greater engagement across the Faculty and reenergised both staff and students around the rewards of working in the helping professions.
